# Effect of Serotype on Focus and Mortality of Invasive Pneumococcal Disease: Coverage of Different Vaccines and Insight into Non-Vaccine Serotypes

**DOI:** 10.1371/journal.pone.0039150

**Published:** 2012-07-16

**Authors:** Albert Jan van Hoek, Nick Andrews, Pauline A. Waight, Robert George, Elizabeth Miller

**Affiliations:** 1 Immunisation, Hepatitis and Blood Safety Department, Health Protection Agency, London, United Kingdom; 2 Statistics Unit, Health Protection Services, Health Protection Agency, London, United Kingdom; 3 Respiratory and Systemic Infection Laboratory, Microbiology Services Division, Health Protection Agency, London, United Kingdom; University Complutense, Spain

## Abstract

**Background:**

Differences in pathogenicity between pneumococcal serotypes are important when assessing the potential benefit of different valency vaccines. We investigated the effect of serotype on clinical presentation, outcome, and quality of life lost from invasive pneumococcal disease (IPD) in the context of the 7, 10, and 13 valent pneumococcal conjugate vaccines (PCV7, PCV10, PCV13).

**Method:**

Serotyped IPD cases in England were linked to the national dataset of hospital admissions for April 2002 to March 2011. Based on patients’ diagnostic codes and vital status at the end of the admission, disease focus (meningitis, empyema, sepsis, or respiratory disease) and case fatality rates by serotype and age group (5, 5–64, and 65 years and over) were obtained. Using these data the quality adjusted life years (QALY) lost from the IPD remaining when use of PCV7 stopped in 2010 was estimated for the serotypes covered by higher valency vaccines.

**Results:**

The linked dataset contained 23,688 cases with information on diagnosis, mortality, and serotype. There were significant differences between serotypes in the propensity to cause meningitis, death, and QALY loss in each of the investigated age groups. As a result, vaccines’ coverage of disease burden differed by endpoint. For example, in children under 5 years in 2009/10, PCV10 covered 39% of meningitis, 19% of deaths and 28% of the QALY loss of attributable to IPD, whereas the respective percentages for PCV13 were 65%, 67%, and 66%. The highest QALY loss per serotype in this age group was for 6A. Non-PCV serotypes causing the highest QALY loss were 22F and 33F in <5 year olds and 31 in older individuals.

**Conclusion:**

Marked differences exist between serotypes in clinical presentation and outcome, and these should be considered when evaluating the potential impact of higher valency vaccines on overall disease burden and associated QALY loss.

## Introduction

*Streptococcus pneumoniae* is a commonly carried bacterium that causes both invasive and non-invasive disease. There are 90+ known serotypes of *S. pneumoniae*, each characterised by the molecular structure of its polysaccharide capsule [1]. Since capsular differences between serotypes have been linked to such properties as carriage prevalence [2], [3], propensity to cause invasive disease [4],[5],[2], and case fatality [6]–[8], each serotype could theoretically be regarded as a separate pathogen [4]. Differences in the pathogenicity and thus clinical impact of different serotypes are important from a public health perspective because available vaccines are serotype-specific, and only target a small subset of the known serotypes. Introduction of the first pneumococcal conjugate vaccine that protected against seven of the most common serotypes in developed countries (PCV7) had a profound effect on serotype-specific carriage prevalence and caused replacement of vaccine types by serotypes not included in the vaccine [9], [3], [10], [11]. The overall impact of this change in carriage prevalence on pneumococcal-attributable morbidity and mortality is dependent on the inherent pathogenicity of the replacing serotypes compared with the previously predominant vaccine types.

**Figure 1:**
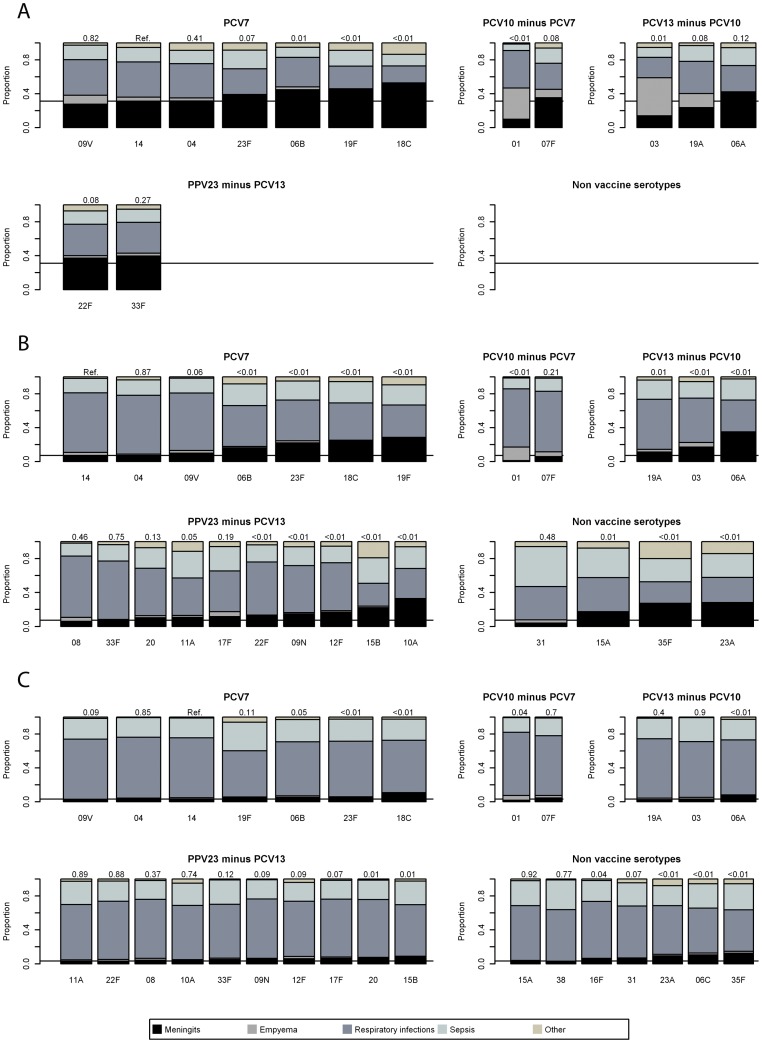
The distribution of disease focus (meningitis, Empyema, respiratory diseases, sepsis and other) per serotype grouped by vaccine type and age group. The line shows the absolute percentage of patients with meningitis for serotype 14, which was used as reference in the logistic regression. The p-values of this regression are shown above each bar. When there are no serotypes with ≥50 cases an empty plot is shown. a) Under 5 years; b) 5–64 years; c) 65 years and older.

To evaluate the potential benefit of introducing higher valency conjugate vaccines, as well as assessing the potential impact of previously uncommon and less studied emerging serotypes, it is important to have information on the invasiveness potential and clinical impact of different serotypes. The latter would ideally include disease focus (eg meningitis, empyema or sepsis), risk of long term sequelae and life years lost as a result of the infection. Expression of the serotype-specific clinical impact in terms of quality of life endpoints would incorporate these multiple facets of disease burden and provide a measure to use in cost-utility evaluations of different intervention strategies, for example, replacing of PCV7 by newer 10 valent (PCV10), 13 valent (PCV13) or higher valency conjugate vaccines.

**Figure 2:**
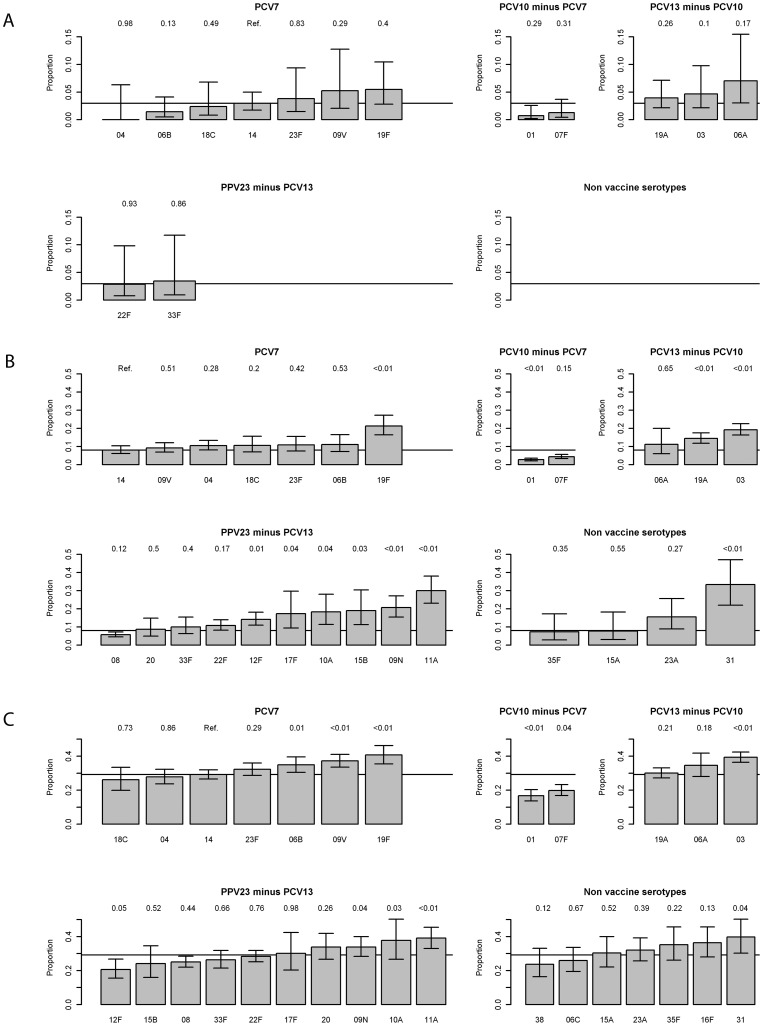
Serotype-specific differences in mortality between serotypes. The line shows the absolute case fatality rate for serotype 14, which was used as reference in the logistic regression. The p-values of this regression are shown above each bar. The whiskers show the 95% confidence intervals based on a binomial distribution. When there are no serotypes with ≥50 cases an empty plot is shown. a) Under 5 years; b) 5–64 years; c) 65+ years.

The aim of this study was to investigate serotype-specific differences in clinical presentation of IPD and impact on quality of life in the context of the newly available PCV vaccines and the existing 23 valent polysaccharide vaccine (PPV23) which, although covering a higher number of serotypes, is poorly immunogenic in children and largely used in risk groups and the elderly. The Health Protection Agency holds one of the largest datasets of invasive pneumococcal disease (IPD) in the world [12] with an annual total of nearly 5000 cases in England and Wales serotyped in recent years. This national dataset has been used to monitor vaccine effects such as herd immunity and serotype replacement after the introduction of PCV7 in 2006 as a 2+1 infant programme [11] and has provided an early indication of the direct effect of PCV13 introduced in April 2010 [13]. Its availability provides a unique opportunity to document the effect on clinical presentation and quality of life of the different serotypes causing IPD over a nine year period spanning the introduction of PPV23 for all 65+ year olds, and the universal infant PCV7 and PCV13 vaccination programmes.

## Methods

### Construction of the Dataset

Microbiology laboratories in England and Wales voluntarily report electronically all clinically significant pneumococcal isolates (obtained by culturing or DNA based methods) to the Health Protection Agency (HPA) [11] and are actively requested to refer these isolates to the Respiratory and Systemic Infection Laboratory (RSIL) for serotyping. Isolates referred to RSIL are confirmed as pneumococci and serotyped with antisera (Statens Serum Institut, Copenhagen) using standard methods. IPD reports with the same serotype within 30 days in the same individual are regarded as the same episode. As the clinical detail routinely available for IPD cases in the national dataset is limited, more comprehensive information on disease focus and outcome by serotype was obtained by linking the laboratory confirmed IPD cases in England (excluding IPD cases from Wales) with the dataset of hospital episode statistics (HES; Copyright © 2012, Re-used with the permission of The Health and Social Care Information Centre. All rights reserved) which is only available for England (10), using National Health Service (NHS) number or a full match on date of birth, sex and postcode. In HES, information on the duration, diagnoses (coded according to the International Classification of Disease series 10, ICD10), operative procedures and deaths during admission are recorded. All admissions with a code specific for IPD, or disease presentations which are likely to be related to acute pneumococcal disease were extracted from the HES database for the administrative years April 2002 to March 2011. The list of ICD10 codes used in the extraction can be found in Supplementary Material S1. Hospital admissions which were on-going the week before and up to a month after the date of the positive culture were included in the analysis. Linkage and subsequent analysis was performed in R 2.12.0 (www.R-project.org).

**Figure 3:**
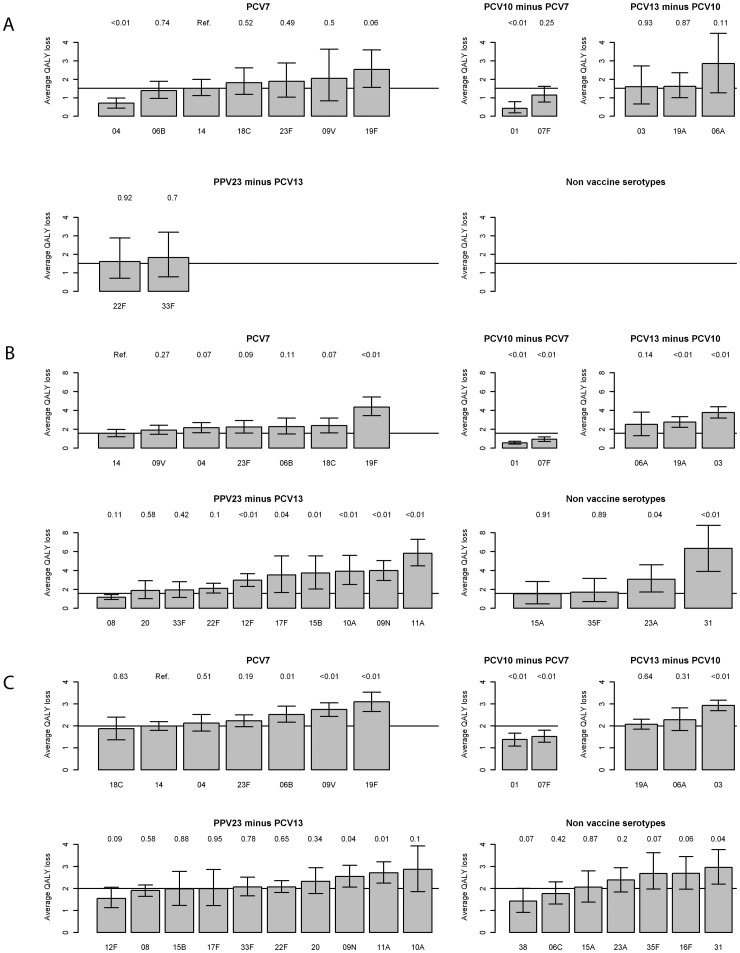
Serotype specific differences in QALY loss (discounted – see text) between serotypes and age groups. The line shows the absolute QALY loss for serotype 14, which was used as reference. The p-values of the bootstrap comparison are shown above each bar. The whiskers show the 95% CI based on 1000 bootstrap samples of the mean. When there are no serotypes with >50 cases an empty plot is shown. a) Under 5 years; b) 5–64 years; c) 65 and over.

### Classification of Disease Focus and Impact on Quality of Life

Disease focus was established using the clinical classifications published by the Healthcare Cost and Utilization Project [14] with some minor adjustments; convulsions (ICD-10 “R56”) were included in the meningitis classification, empyema was included as a separate condition based on ICD-10 code “J86” and the different respiratory classifications (pneumonia, lower and upper respiratory tract infections) were grouped into one broad respiratory disease category (excluding empyema). Categorisation was based on all ICD-10 diagnosis codes for an admission rather than just the primary diagnosis, thus highlighting the most important focus of the pneumococcal-related infection. Where patients had multiple pneumococcal related diagnoses the most severe was chosen in the order; meningitis, empyema, sepsis, respiratory disease and other. For the serotype-specific analyses only serotypes isolated from at least 50 episodes of IPD were included. Cases for which only the serogroup was known, and cases serotyped as 6A but not tested for 6C were excluded from the analysis.

Mortality was based on the discharge information within HES. Only deaths within 30 days of the culture date were regarded as pneumococcal-attributable. As serotype distribution, disease presentation, and mortality varies between age-groups, data are presented for the age groups 0–4 years, 5–64 and 65+ years.

**Table 1:** Cumulative proportion of meningitis, mortality and QALY loss (discounted) in 2009/2010 in England attributable to invasive pneumococcal disease covered by different valency vaccines.

Cumulative contribution		PCV7*	PCV10**	PCV13***	PPV23	Remaining % Non-Vaccine types
<5 year olds	IPD	5%	41%	75%	90%	10%
	Meningitis	8%	39%	65%	88%	12%
	Mortality	6%	19%	67%	85%	15%
	QALY	7%	28%	66%	86%	14%
	QALY (disc)	7%	28%	66%	86%	14%
5–64 year olds	IPD	10%	43%	61%	90%	10%
	Meningitis	14%	24%	49%	78%	22%
	Mortality	12%	23%	52%	86%	14%
	QALY	12%	25%	52%	86%	14%
	QALY (disc)	12%	24%	52%	86%	14%
≥65 year olds	IPD	12%	23%	51%	81%	19%
	Meningitis	11%	19%	38%	65%	35%
	Mortality	13%	21%	53%	81%	19%
	QALY	13%	21%	53%	81%	19%
	QALY (disc)	13%	21%	53%	81%	19%

* Contains serotypes 4,6B,9V,14,18C,19F,23F.

** Contains additional serotypes 1,5,7F.

*** Contains additional serotypes 3, 6A,19A.

To compare the loss in quality adjusted life years (QALYs) by serotype an average QALY loss per case of 0.0079 was assumed for hospitalisation with a non-meningitis focus [15], 0.023 QALY loss for hospitalisation due to meningitis [15] with a further 0.255 QALY loss for each remaining life year applied to the 31.7% of meningitis cases expected to have long term sequelae [16]. Empyema is severe in the acute phase [17], and in absence of a published QALY loss estimates for empyema, the QALY loss for meningitis was applied. No QALY loss from sequelae of empyema was included as the long term outcome is good [17]. For fatal cases, we used one QALY for each lost life year as expected by the gender specific life expectancy which is based on the 2010 mortality rates for England [18]. When conducting economic analyses future disease burden is normally discounted to reflect a time preference, therefore we added a discount rate of 3.5% per annum, as recommended by the National Institute for Health and Clinical Excellence (NICE) [19].

### Statistical Analysis

To assess differences between serotypes we calculated the odds ratio of developing meningitis and death for a given serotype compared to serotype 14. This is because serotype 14 had the greatest number of samples, and has previously been used as a reference for intra-serotype comparisons [7]. To correct for potential confounding due to age (years), gender, socio-economic factors, co-morbidities, study year and alcoholism we used a binomial logistic regression for meningitis and mortality. Due to the bimodal distribution of the QALY loss we show a p-value based on the difference of 1000 bootstrap samples for the mean of the given serotype and serotype 14 (this approach precluded correction based on potential confounders). For all tests the (adjusted) p-values are presented in the paper and the obtained odds are included in Supplementary Material S1. Socio-economic deprivation was based on the rank in the deprivation index, as published by the Department for Communities and Local Government in the UK [20]. This index is assigned to a small geographical area (lower super output area) and related to the postcode of the patient at the time of admission as recorded in HES. The deprivation index is not updated every year so to reduce the effect of changes of deprivation over time we divided the rank into quartiles, as it is less likely that a neighbourhood will change so extensively that it moves over quartiles. Co-morbidities were scored based on the Charlson index, where the included conditions were crossed mapped with ICD-10 codes [21]. In the analysis the Charlson index was sub-grouped into “no-comorbidities”, “Charlson score 1–2” and “3 and above”. As alcoholism is not a part of the Charlson index patients were identified for alcohol related problems based on the codes used by Harboe et al [6].

To assess the precision of the estimates, binomial confidence intervals are presented for mortality and the 2.5% and 97.5% percentiles of 1000 bootstrap samples for the mean QALY loss.

To investigate the proportion of IPD (and its various disease outcomes) that was possibly preventable by the different pneumococcal vaccines (PCV7, PCV10, PCV13 and PPV23) at the time PCV13 was introduced in April 2010, the number of cases by age group and clinical endpoint was estimated for each serotype. This was achieved by multiplying the serotype specific percentage with meningitis, the case fatality rate (CFR) or QALY loss as measured over the full period with the absolute serotype distribution in the period April 2009 to March 2010. In this calculation the serotypes with less than 50 cases were included.

## Results

### Linkage Success

The linkage was increasingly successful over the years, with 50% of IPD cases linked to a HES admission in 2002/2003 rising to 76% in 2010/2011, resulting in a total of 33,196 linked cases over the nine year period from England. Of these, 23,688 (71%) had information on ICD-10 diagnoses, mortality and infecting serotype (2,605, 10,389 and 10,694 for the age groups <5,5–65 and 65 and over). The matched cases had a similar serotype and age distribution to the unmatched cases, suggesting that there were no major biases with respect to these variables as a result of the incomplete linkage (see the figures in Supplementary Material S1).

**Table 2:** Number of cases of IPD, meningitis, deaths and the total QALYs lost by age group in England 2009/2010.

	Number of cases		Number of meningitis cases		Number of deaths		QALYs lost (undiscounted)		QALYs lost (discounted)	
Age	Total	%	total	%	total	%	total	%	Total	%
0–4	572	10%	161	27%	17	2%	2414	13%	831	7%
5–64	2735	48%	310	53%	277	27%	9646	52%	5589	48%
65+	2413	42%	119	20%	716	71%	6394	35%	5218	45%
Total	5719	100%	591	100%	1010	100%	18454	100%	11638	100%

For the under 5 year olds there was a total of 51 different serotypes in the matched dataset; however many did not achieve the pre-specified minimum of 50 cases leaving only 14 serotypes (comprising 86% of the cases) for analysis. Among 5–64 year old patients, 67 different serotypes were identified, with only 26 (comprising 96% of the cases) having enough cases for individual analysis. Among the 65 years and over, 62 different serotypes were recorded, with 29 (comprising 97% of the cases) having enough for individual analysis.

### Serotype- Specific Disease Focus

Within each age group, the clinical presentation differed significantly between serotypes, even after correcting for co-morbidities and socio-economic factors, see Figure 1 and Supplementary Material S1. Among children under 5 years, serotype 18C was the most likely to cause meningitis, with 52% of the patients presenting with this outcome, followed by 19F (46%) and 6B (45%). All three serotypes were significantly more likely to cause meningitis compared to serotype 14 (31%). The non-PCV serotypes, 22F and 33F, had a percentage with meningitis of 37% and 40% respectively. The lowest proportion of meningitis cases was among serotypes 1 (10%), 3 (14%) and 19A (24%), although only serotype 1 and 3 were significantly different from serotype 14. The serotypes causing a low burden of meningitis had a relatively high percentage of children presenting with empyema; serotype 3 (45%), 1 (37%) and 19A (17%) showed the highest proportions with empyema whereas only 0–3% of the serotypes associated with meningitis (6A, 19F, 18C 22F and 33F) caused empyema. The percentage of IPD resulting in meningitis was lower in the age group 5–64 years than in the under 5 year olds (10% vs 33%). Compared to serotype 14 (7% with meningitis in this age group), serotypes 6A (34%), 10A (32%), and 23A (28%) were significantly more likely to cause meningitis, as were 6B, 19F, 18C, 35F and 15B. Serotype 1 rarely caused meningitis (1%), followed by 31 (4%) and 7F (6%). Again serotype 1 was linked to empyema, with 16% of cases having this presentation, followed by 7F (6%). Serotypes more likely to cause meningitis rarely caused empyema - 6A (1%), 10A (1%) and 23A (0%).

For the age group 65 years and over few cases presented with meningitis or empyema. The serotypes most likely to cause meningitis were 35F (13%), 6C (10%) and 18C (10%). For empyema, serotype 1 showed the strongest association (6%) followed by 7F and 12F (both 3%).

In the regression analysis, having no co-morbidity was significantly associated with a higher probability of developing meningitis in the patients above 5 years of age (odds 1.88 and 2.51 respectively). The other confounders were not significantly associated with meningitis and/or no trends by year or socio-economic factors were identified. Disease focus over time (results not shown) was stable.

### Serotype-specific Mortality

The case fatality among under 5 year olds was low (overall 3%). The serotype with the highest case fatality rate was 6A with 7%, followed by 19F with 5%, and 9V and serotype 3 (5%) (see Figure 2 and Supplementary Material S1). Serotype 4 (0%) had the lowest case fatality rate followed by serotypes 1, 7F and 6B each with 1%. However, none of these case fatality rates differed significantly from serotype 14 (3%). Non PCV types 22F (3%) and 33F (3%) had an average mortality.

Among the age group 5–64, (overall CFR 10%) serotypes 31 (33%), 11A (30%) and 19F (21%) had the highest case fatality rates; serotypes 1 (3%), 7F (4%) and 8 (6%) had the lowest rates. Serotype 3, 19F, 19A, 6A, 9N, 11A and 31 (the last 3 serotypes are not included in any current conjugated vaccine), were all significant higher compared to serotype 14 (8%), only serotype 1 was significantly lower.

Patients aged 65 years and over had the highest case fatality rate (overall 30%). Serotypes most likely to be associated with a fatal outcome were 19F (41%), 31 (40%), and 3 (39%), all significantly different from serotype 14 (29%). The lowest case fatality rates were for serotypes 1 (17%), 7F (20%) and 12F (21%), all three significantly lower than serotype 14.

Confounders associated with mortality in all age groups were meningitis (p value: <0.01; <0.01; 0.03 for the age groups <5, 5–64 and 65+ years respectively), and co-morbidities (p values: 0.06; <0.01, <0.01 respectively). Among the age group 5–64 years there was a decline in mortality over time (reducing the odds to 0.6 in 2010–2011 compared to 2002–2003) and a declining risk of mortality by declining social-economic deprivation (an odds of 0.7 in the least deprived status compared to the most deprived). These trends were not observed for the other age groups.

### QALY Loss by Serotype

The lowest overall QALY loss per serotype among children under 5 years was serotype 1 (0.43 per case) followed by 4 (0.71) and 7F (1.15). The highest QALY loss was among serotypes 6A (2.86). 19F (2.53) and 9V (2.05). Serotype 22F scored 1.62 and 33F 1.83 (see Figure 3 and Supplementary Material S1for more detail).

Within the age group 5–64 years the difference between the low and high burden of disease serotypes was more marked. The serotypes causing the highest QALY loss were 31 (6.34), 11A (5.82) and 19F (4.34). Serotype 1, 7F and 8 had the lowest QALY loss 0.57, 0.95 and 1.17 respectively.

Among the 65 years and over the differences between serotypes in QALY loss per case declined again, with serotypes 1 (1.38), 38 (1.42), and 7F (1.52) at the low end of the spectrum and 19F (3.09), 31 (2.95) and 3 (2.93) on the high end.

We performed a sensitivity analysis (results not shown) because a significant decrease of mortality was observed for 5–64 years over the period. We adjusted the QALY loss for each death before July 2006 by replacing it by p*(QALY loss for death) + (1-p)*(multiplication with the odds for mortality in the second half of the period (July 2006 onwards) and adding the QALY loss for meningitis) where p was the adjusted odds ratio of mortality after July 2006 compared to before July 2006 by age. Doing so resulted in slightly lower QALY losses, and only minor changes in the ranking of serotypes.

### Burden of IPD Potentially Preventable by Different Valency Vaccines in England before PCV13 Introduction

The contribution of the vaccine-specific serotypes to the overall burden of IPD in England changed over the study period, largely due to the impact of PCV7 on serotype distribution. For 2009/2010, the last administrative year before PCV13 introduction, the contribution of the different vaccine-specific serotype groupings to the overall burden of IPD and its associated QALY loss are shown in Table 1 by age group. For children under 5 years, the cumulative coverage for PCV10 was 41% for all IPD, 39% for meningitis and 19% for mortality, and 28% for the total QALYs lost. For PCV13 the coverages were 75%, 65%, 67% and 66% respectively for the same outcomes. PCV13 therefore covered 1.8 times more IPD cases compared to PCV10, 1.6 times more meningitis, 3.5 times more mortality, and 2.4 times the total QALYs lost. For patients 5–64 years, PCV13 covered 1.4 times more cases of IPD, 2 times more cases of meningitis, 2.2 times more cases of mortality and 2.1 times as much QALY loss as PCV10. Among the 65 years and over, PCV13 covered 2.2 times more IPD cases compared to PCV 10, 2.0 times more meningitis cases, 2.6 times more fatal cases and 2.5 times the number of QALYs lost.

### Deaths and Life Years Lost due to IPD, England 2009/2010

In 2009/2010 there were 5,719 cases of IPD confirmed by culture or polymerase chain reaction in the England IPD dataset. Based on the age-specific serotype distribution in that year and the disease focus and outcome for each serotype averaged over the nine year study period, there was an estimated total of 591 meningitis cases and 1,010 deaths in 2009/2010 year attributable to IPD, with an associated QALY loss of 18,454 (11,638 discounted) in England. The contribution of different age groups to these cumulative totals varied considerably (Table 2). The main burden of meningitis was among the young and the middle age group, but the majority of deaths were among the ≥ 65year olds (71%). For QALY loss (undiscounted), most of the burden was in the age group 5–64, though after discounting their QALY loss was similar to that in the ≥ 65 year age group.

## Discussion

Our study documents the clinical presentation, mortality and impact on the quality of life of the prevalent pneumococcal serotypes causing IPD in England in recent years. The serotype-specific clinical presentations were broadly stable over time, consistent with reflecting an inherent property of each serotype. To our knowledge, this is the first attempt to compare disease outcome between serotypes based on QALY loss and incorporating acute disease burden, long term sequelae, mortality and life years lost. Our results add to the understanding of the role of the capsular type of *S. pneumoniae* in determining pathogenicity and can guide decision making on the potential health gain of introducing vaccines with improved serotype coverage.

Assigning QALY weights to different disease states is a well-established approach for comparing the potential health gain of different therapeutic or prophylactic interventions as it combines both duration and quality of life in a single measure. While for non-fatal diseases QALY estimation can be problematic because of reliance on subjective measures, for IPD the QALY differences between serotypes are mainly driven by the life years lost – a more objective measure. We believe QALY loss estimates provide a better platform to distinguish between serotypes causing a low and high disease burden than simply reporting mortality or meningitis rates. Expressed in QALYs the main IPD burden was found amongst 5–64 year olds, where the higher number of life years lost outweighed the higher case fatality rate among the ≥ 65 year olds with their lower life expectancy.

Serotypes with a high and low case fatality rate in our study were the same serotypes found to be linked to a high and low case fatality rate in a study in Denmark [6] and in a review on mortality by capsular type that included data from 9 different studies from the United States, Europe, Africa and the Middle East spanning the period 1952 to 2010 [22]. This supports the view that high or low mortality is a stable feature for those serotypes, though there was less consistency between the studies for the serotypes which were not on the extremes. Our results show that the differences between serotypes are most marked in the age group 5–64 years. This may reflect the greater vulnerability of the very young and elderly populations to IPD which may in part mask the inherent differences between serotypes.

It is not clear how the capsular differences between serotypes affect clinical presentation and outcome (22). Differences in capsular size or molecular structure could possibly lead to a different interaction of the bacteria with its environment (including other bacteria in the nasopharynx) and/or immune system in the blood, brain or other tissues. Although the capsule is the major virulence factor [23], there are other factors such as surface proteins and enzymes, and the major toxin pneumolysin, which determine virulence. If these non-capsular virulence factors are also associated specifically with certain serotypes then some of the characteristics attributed to differences in serotype *per se* may be spurious. Whole genome sequencing has also identified a number of highly variable pathogenicity islands within the pneumococcal genome, with considerable variation between strains. Some serotypes are highly clonal while others exhibit considerable genetic diversity [24]. The extent to which genetically diverse strains within the same serotype exhibit different behaviour in terms of clinical presentation and outcome cannot be assessed by our study and would require parallel genetic information. Another caveat in attributing the observed characteristics to specific serotypes is that differentiation into serotypes within a serogroup is still evolving as shown for 6A for which the original serotyping methods failed to distinguish to 6A from 6C [25], each with a different clinical outcome.

Host factors can also affect clinical presentation, as shown by the lower propensity to develop meningitis in individuals aged over 5 years with co-morbidities. In addition, factors such as ethnicity, socio-economic or other environmental factors could influence disease focus as suggested by the strong association between serotype 1 and meningitis in west-Africa. In our study, as in a hospital-based study in Spain [26], serotype 1 was predominantly associated with empyema. Therefore caution should be exercised in translating our results to all epidemiological settings.

Our study has the potential limitation that we were not able to link all cases in the national IPD dataset with a HES admission. Failure to match could be due to an incomplete initial extract from the HES database omitting relevant diagnoses that could denote IPD, or be due to non-hospitalised invasive disease or incomplete information in the fields used in matching. Thus there is the possibility of a selection bias, excluding certain clinical presentations or more mild disease. However the similarity between the linked and the unlinked dataset in age and serotype distribution suggests that the linked subset is representative of the complete dataset.

From our analysis PCV13 is predicted to protect against a substantially greater burden of invasive disease than PCV10, especially mortality, based on its additional coverage of serotypes 3, 6A and 19A, and the serotype distribution in 2009 in England. However when deciding between the two vaccines, additional factors need to be taken into account. These include the serotype distribution and burden of non-invasive disease, the potential to prevent disease due to non-typeable *Haemophilus influenza* with PCV10 (which is conjugated to *Haemophilus influenzae* protein-D [27]) and indirect effects due to differences in carriage prevalence of the serotypes covered by each vaccine; for example the extra three serotypes in PCV10 compared with PCV7 (1, 5 and 7F) have a very low carriage prevalence, due to this low carriage there is potential less space for replacement disease by non-vaccine types compared to the extra serotypes in PCV13, which are more prevalent in carriage [3]. The overall impact on the burden of IPD of each vaccine may therefore be affected by the potential for serotype replacement and the invasiveness of the replacing strains [3]. For the emerging non PCV serotypes 11A, 31, 10A and 9N could be prioritised for inclusion in future conjugated vaccines, as they have a relatively high QALY loss.

In conclusion, from our large linked dataset with information on serotype and clinical outcome, we were able to confirm marked and stable differences in morbidity and mortality between pneumococcal serotypes, provide estimates for the proportion of cases by age group and serotype with meningitis, empyema and mortality, and derive the estimated annual QALY loss from IPD four years after the introduction of PCV7 in England. While many of the clinical outcomes seem to be robustly linked to the capsular type, extrapolation of our findings to populations with vastly different epidemiological and socio-economic backgrounds should be done carefully. Our findings have relevance for future work on capsular differences and interaction with the host immune system, and can inform decision modelling of the relative merits of vaccines with different serotype-composition.

### Ethics Approval

The Health Protection Agency has approval under PIAG Section 60 of the Health and Social Care Act 2001(now subsumed into the National Information Governance Board for Health and Social Care with Section 60, now Section 251 of the NHS Act 2006) to process confidential patient information for the purposes of monitoring the efficacy and safety of vaccination programmes.

## Supporting Information

Supplementary Material S1 The supplementary material contains the list of ICD-10 codes used in the data extraction, a comparison between the matched and non-matched dataset, and the serotype specific outcomes (by age group) and outcomes from the regression (by age group).
(DOCX)

## References

[1] Bentley SD, Aanensen DM, Mavroidi A, Saunders D, Rabbinowitsch E (2006). Genetic analysis of the capsular biosynthetic locus from all 90 pneumococcal serotypes.. PLoS genetics.

[2] Sleeman KL, Griffiths D, Shackley F, Diggle L, Gupta S (2006). Capsular serotype-specific attack rates and duration of carriage of Streptococcus pneumoniae in a population of children.. The Journal of infectious diseases.

[3] Flasche S, Van Hoek AJ, Sheasby E, Waight P, Andrews N (2011). Effect of pneumococcal conjugate vaccination on serotype-specific carriage and invasive disease in England: a cross-sectional study.. PLoS medicine.

[4] Scott JA, Hall AJ, Dagan R, Dixon JM, Eykyn SJ (1996). Serogroup-specific epidemiology of Streptococcus pneumoniae: associations with age, sex, and geography in 7,000 episodes of invasive disease.. Clinical infectious diseases.

[5] Brueggemann AB, Griffiths DT, Meats E, Peto T, Crook DW (2003). Clonal relationships between invasive and carriage Streptococcus pneumoniae and serotype-and clone-specific differences in invasive disease potential.. Journal of Infectious Diseases.

[6] Harboe ZB, Thomsen RW, Riis A, Valentiner-Branth P, Christensen JJ (2009). Pneumococcal serotypes and mortality following invasive pneumococcal disease: a population-based cohort study.. PLoS medicine.

[7] Weinberger DM, Harboe ZB, Sanders EAM, Ndiritu M, Klugman KP (2010). Association of serotype with risk of death due to pneumococcal pneumonia: a meta-analysis.. Clinical infectious diseases.

[8] Jansen AGSC, Rodenburg GD, van der Ende A, van Alphen L, Veenhoven RH (2009). Invasive pneumococcal disease among adults: associations among serotypes, disease characteristics, and outcome.. Clinical infectious diseases.

[9] Cheung Y-B, Zaman SMA, Nsekpong ED, Van Beneden CA, Adegbola RA (2009). Nasopharyngeal carriage of Streptococcus pneumoniae in Gambian children who participated in a 9-valent pneumococcal conjugate vaccine trial and in their younger siblings.. The Pediatric infectious disease journal.

[10] Spijkerman J, van Gils EJM, Veenhoven RH, Hak E, Yzerman F (2011). Carriage of Streptococcus pneumoniae 3 Years after Start of Vaccination Program, the Netherlands.. Emerging Infectious Diseases.

[11] Miller E, Andrews NJ, Waight PA, Slack MP, George RC (2011). Herd immunity and serotype replacement 4 years after seven-valent pneumococcal conjugate vaccination in England and Wales: an observational cohort study.. The Lancet infectious diseases.

[12] World Health Organisation (2011). Review of Serotype Replacement in the Setting of PCV7 Use and Implications for the PCV10/PCV13 Era.. http://www.who.int/immunization/sage/SAGEReplacementReport2011FINAL_nov11.pdf.

[13] Miller E, Andrews NJ, Waight PA, Slack MPE, George RC (2011). Effectiveness of the new serotypes in the 13-valent pneumococcal conjugate vaccine.. Vaccine.

[14] Healthcare cost and Utilization Project (2012). Clinical classifications.. http://www.hcup-us.ahrq.gov/toolssoftware/icd_10/ccs_icd_10.jsp.

[15] Bennett JE, Sumner W, Downs SM, Jaffe DM (2000). Parents’ utilities for outcomes of occult bacteremia.. Archives of pediatrics & adolescent medicine.

[16] Jit M (2010). The risk of sequelae due to pneumococcal meningitis in high-income countries: a systematic review and meta-analysis.. The Journal of infection.

[17] Sawicki GS, Lu FL, Valim C, Cleveland RH, Colin AA (2008). Necrotising pneumonia is an increasingly detected complication of pneumonia in children.. The European respiratory journal.

[18] National Office for Statistics (2011). Interim Life Tables, 2008–2010.. http://www.ons.gov.uk/ons/rel/lifetables/interim-life-tables/2008-2010/rft-ilt-ew-2008-2010.xls.

[19] (2008). Guide to the methods of technology appraisal. London: National Institute for Health and Clinical Excellence.. 76 p.

[20] Department for Communities and Local Government (2011). The English Indices of Deprivation.. http://www.communities.gov.uk/publications/corporate/statistics/indices2010.

[21] Sundararajan V, Henderson T, Perry C, Muggivan A, Quan H (2004). New ICD-10 version of the Charlson comorbidity index predicted in-hospital mortality.. Journal of clinical epidemiology.

[22] Weinberger DM, Harboe ZB, Sanders E a M, Ndiritu M, Klugman KP (2010). Association of serotype with risk of death due to pneumococcal pneumonia: a meta-analysis.. Clinical infectious diseases.

[23] AlonsoDeVelasco E, Verheul A, Verhoef J, Snippe H (1995). Streptococcus pneumoniae: virulence factors, pathogenesis, and vaccines.. Microbiological reviews.

[24] Elberse KEM, van de Pol I, Witteveen S, van der Heide HGJ, Schot CS (2011). Population structure of invasive Streptococcus pneumoniae in The Netherlands in the pre-vaccination era assessed by MLVA and capsular sequence typing.. PloS one.

[25] Park IH, Pritchard DG, Cartee R, Brandao A, Brandileone MCC (2007). Discovery of a new capsular serotype (6C) within serogroup 6 of Streptococcus pneumoniae.. Journal of clinical microbiology.

[26] Burgos J, Lujan M, Falcó V, Sánchez A, Puig M (2011). The spectrum of pneumococcal empyema in adults in the early 21st century.. Clinical infectious diseases.

[27] GlaxoSmithKline Biologicals (2011). Package insert Synflorix. 9.. http://www.who.int/immunization_standards/vaccine_quality/Synflorix_WHO_leaflet_EN_May_2011.pdf.

