# Supplementary material for: Effect of Serotype on Focus and Mortality of Invasive Pneumococcal Disease: Coverage of Different Vaccines and Insight into Non-Vaccine Serotypes
Source: PLoS One. 2012 Jul 16;7(7):e39150. doi: 10.1371/journal.pone.0039150 (PMC3398022; doi:10.1371/journal.pone.0039150)
Supplement: Supplementary Material S1 — The supplementary material contains the list of ICD-10 codes used in the data extraction, a comparison between the matched and non-matched dataset, and the serotype specific outcomes (by age group) and outcomes from the regression (by age group). (DOCX) [file pone.0039150.s001.docx]

Supplementary material

List of ICD-10 codes used in the initial extraction from the Hospital Episode Statistics database.

| ICD10 code | Description |
| --- | --- |
| A40.3 | Septicaemia due to Strep Pneumoniae |
| A40.8 | Other streptococcal septicaemia |
| A40.9 | Strep. Septicaemia non specified |
| A41.9 | Septicaemia, unspecified |
| A49.1 | Streptococcal unspecified |
| A49.9 | Bacterial infection, unspecified |
| B95.3-5 | Step pneum as the cause of disease classified in other chapters |
| G00.1 | Pneumococcal meningitis |
| G00.8 | Other bacterial meningitis |
| G00.9 | Bacterial meningitis unspecified |
| G04.2 | Bacterial meningoencephalitis and meningomyelitis not elsewhere classified |
| G04.8 | Other encephalitis, myelitis and encephalomyelitis |
| G04.9 | Encephalitis, myelitis and encephalomyelitis, unspecified |
| G05.0 | Encephalitis, myelitis and encephalomyelitis in bacterial diseases classified elsewhere |
| H67 | Otitis Media |
| I33.0 | Acute and subacute infective endocarditis |
| J00 | Acute Nasopharyngitis |
| J01 | Acute sinusitis |
| J02.0 | Acute pharyngitis Streptococcal |
| J02.9 | Acute pharyngitis, unspecified |
| J03.0 | Streptoccocal tonsillitis |
| J03.9 | Tonsillits unspecified |
| J04 | Acute Laryngitis and tracheitis |
| J05 | Acute obstructive laryngitis |
| J06 | Acute upper respiratory infections of multiple and unspecified sites |
| J13 | Pneumonia due to Streptococcus pneumoniae |
| J15.9 | Bacterial pneumonia, unspecified |
| J18 | Pneumonia, organism unspecified |
| J20.2 | Acute Bronchitis due to streptococcus |
| J20.9 | Acute bronchitis, unspecified |
| J21.8 | Acute Bronchiolitis other specified |
| J21.9 | Acute Bronchiolitis, unspecified |
| J22 | Unspecified acute lower respiratory infection |
| J40 | Bronchitis, not specified as acute or chronic |
| J42 | Unspecified chronic bronchitis |
| J86 | Pyothorax |
| M00.1 | Pneumococcal arthritis and polyarthritis |
| M00.2 | Other streptococcal arthritis and polyarthritis |
|  |  |
| Alcohol related codes | F10, G31.2, G62.1, G72.1, I42.6, K29.2, K86.0, K70, R78.0, T51, Z72.1 |

Comparison between the matched and non-matched dataset.

Figure S-1 Comparison of the contribution of vaccine types in the unmatched (left) and matched (right) for each year in the dataset.


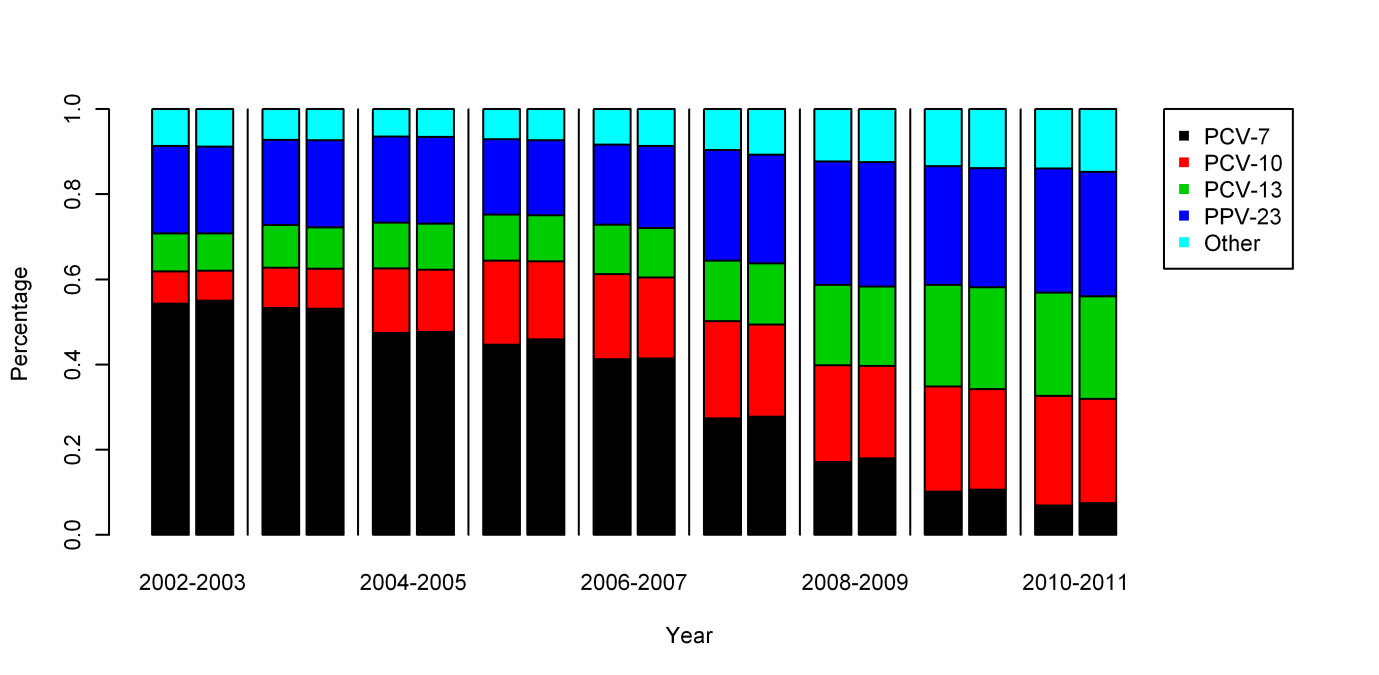


Figure S-2 Comparison of the contribution of age groups to the unmatched (left) and matched (right) for each year in the dataset.


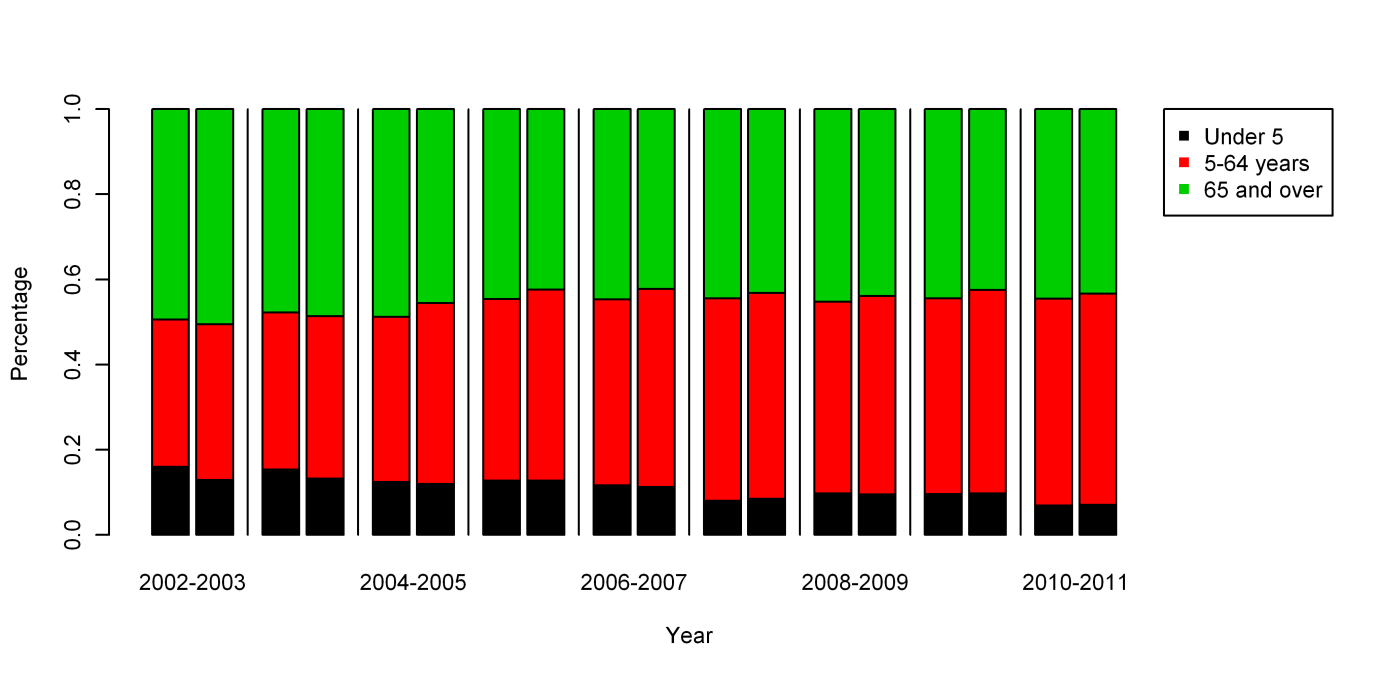


Serotype specific outcomes and outcomes from the regression.

Table 1 Outcomes for the individual serotypes with n>50 for the age group 0-4 years (serotypes not in any of the PCV vaccines are highlighted in bold)

| serotype | n | Meningitis | Empyema | Pneumonia | Septicemia | Other | Odds Meninigits | Mortality | Odds Mortality | QALY (disc) | QALY(undisc) |
| --- | --- | --- | --- | --- | --- | --- | --- | --- | --- | --- | --- |
| 4 | 57 | 32% | 4% | 40% | 16% | 9% | 1.3 (0.69-2.45) | 0% (0%-6.3%) | 0 (0-0) | 0.71 (0.44-0.98) | 2.00 |
| **33F** | 58 | 40% | 3% | 36% | 16% | 5% | 1.42 (0.76-2.65) | 3.4% (1%-11.7%) | 1.16 (0.24-5.72) | 1.83 (0.77-3.29) | 5.30 |
| **22F** | 70 | 37% | 3% | 37% | 16% | 7% | 1.69 (0.93-3.06) | 2.9% (0.8%-9.8%) | 1.08 (0.21-5.47) | 1.62 (0.71-2.92) | 4.70 |
| 6A | 71 | 42% | 0% | 31% | 21% | 6% | 1.54 (0.89-2.68) | 7% (3%-15.4%) | 2.21 (0.71-6.9) | 2.86 (1.3-4.6) | 8.20 |
| 9V | 76 | 28% | 11% | 42% | 17% | 3% | 1.07 (0.6-1.9) | 5.3% (2.1%-12.8%) | 1.91 (0.58-6.3) | 2.05 (0.81-3.57) | 5.80 |
| 23F | 105 | 38% | 1% | 30% | 22% | 9% | 1.54 (0.96-2.49) | 3.8% (1.5%-9.4%) | 1.14 (0.35-3.7) | 1.89 (1.01-2.96) | 5.40 |
| 18C | 125 | 52% | 1% | 20% | 14% | 14% | 3.17 (2.04-4.94) | 2.4% (0.8%-6.8%) | 0.63 (0.17-2.32) | 1.82 (1.18-2.62) | 5.20 |
| 3 | 129 | 14% | 45% | 24% | 12% | 5% | 0.47 (0.26-0.84) | 4.7% (2.1%-9.8%) | 2.51 (0.83-7.56) | 1.6 (0.7-2.74) | 4.70 |
| 19F | 146 | 46% | 0% | 27% | 18% | 9% | 1.96 (1.3-2.95) | 5.5% (2.8%-10.4%) | 1.49 (0.59-3.79) | 2.53 (1.55-3.7) | 7.40 |
| 6B | 210 | 45% | 3% | 35% | 12% | 5% | 1.65 (1.15-2.36) | 1.4% (0.5%-4.1%) | 0.37 (0.1-1.34) | 1.39 (0.99-1.91) | 4.10 |
| 7F | 232 | 35% | 10% | 31% | 18% | 6% | 1.45 (0.96-2.18) | 1.3% (0.4%-3.7%) | 0.49 (0.13-1.91) | 1.15 (0.78-1.63) | 3.30 |
| 19A | 253 | 24% | 17% | 38% | 19% | 3% | 0.69 (0.46-1.04) | 4% (2.2%-7.1%) | 1.74 (0.67-4.52) | 1.62 (0.97-2.34) | 4.70 |
| 1 | 280 | 10% | 37% | 44% | 8% | 1% | 0.42 (0.26-0.69) | 0.7% (0.2%-2.6%) | 0.43 (0.09-2.05) | 0.43 (0.19-0.79) | 1.20 |
| 14 | 439 | 31% | 5% | 41% | 17% | 5% | 1 | 3% (1.7%-5%) | 1 | 1.51 (1.06-1.98) | 4.40 |

Table 2 Outcome regression for other factors as serotype for the age group 0-4 years

|  | Meningitis | Mortality |
| --- | --- | --- |
| Sex |  |  |
| Female | 1 | 1 |
| Male | 1.1 (0.92-1.32) | 0.93 (0.58-1.48) |
| Year |  |  |
| 2002-2003 | 1 | 1 |
| 2003-2004 | 0.69 (0.47-1.03) | 0.75 (0.26-2.17) |
| 2004-2005 | 0.66 (0.45-0.97) | 1.49 (0.59-3.76) |
| 2005-2006 | 0.84 (0.58-1.22) | 0.62 (0.23-1.68) |
| 2006-2007 | 0.57 (0.38-0.84) | 0.88 (0.33-2.34) |
| 2007-2008 | 0.72 (0.46-1.11) | 0.7 (0.23-2.13) |
| 2008-2009 | 0.62 (0.41-0.95) | 0.91 (0.32-2.59) |
| 2009-2010 | 0.59 (0.38-0.91) | 0.72 (0.24-2.17) |
| 2010-2011 | 0.62 (0.38-1.03) | 0.64 (0.17-2.47) |
| Comborbidities |  |  |
| With co-morbidities (Charlson>0) | 1 | 1 |
| No co-morbidities (Charlson = 0) | 0.81 (0.58-1.13) | 0.53 (0.27-1.02) |
| Meningitis |  |  |
| No | NA | 1 |
| Yes | NA | 4.35 (2.57-7.35) |
| Socio-economics |  |  |
| Level 1 | 1 | 1 |
| Level 2 | 1.07 (0.82-1.39) | 0.94 (0.5-1.8) |
| Level 3 | 0.91 (0.69-1.2) | 0.33 (0.12-0.87) |
| Level 4 | 1.19 (0.91-1.56) | 1.07 (0.56-2.05) |
| Level 5 | 1.22 (0.93-1.61) | 0.96 (0.49-1.88) |

Table 3 Outcomes for the individual serotypes with n>50 for the age group 5-64 years (serotypes not in any of the PCV vaccines are highlighted in bold, and serotypes which are not in PCV or PPV vaccines are highlighted in bold and underlined).

| serotype | n | Meningitis | Empyema | Pneumonia | Septicemia | Other | Odds Meningitis | Mortality | Odds Mortality | QALY (disc) | QALY(undisc) |
| --- | --- | --- | --- | --- | --- | --- | --- | --- | --- | --- | --- |
| **31** | 51 | 4% | 4% | 39% | 47% | 6% | 0.59 (0.14-2.53) | 33.3% (22%-47%) | 4.79 (2.38-9.64) | 6.34 (3.84-8.73) | 10.80 |
| **15A** | 52 | 17% | 0% | 40% | 35% | 8% | 3.15 (1.41-7.07) | 7.7% (3%-18.2%) | 0.71 (0.24-2.15) | 1.53 (0.48-2.87) | 2.50 |
| **17F** | 52 | 12% | 6% | 48% | 29% | 6% | 1.85 (0.73-4.64) | 17.3% (9.4%-29.7%) | 2.38 (1.04-5.42) | 3.53 (1.57-5.66) | 6.40 |
| **35F** | 55 | 27% | 0% | 25% | 27% | 20% | 5.41 (2.7-10.86) | 7.3% (2.9%-17.3%) | 0.59 (0.19-1.78) | 1.7 (0.69-2.86) | 2.80 |
| **15B** | 63 | 22% | 2% | 27% | 30% | 19% | 4.1 (2.05-8.18) | 19% (11.2%-30.4%) | 2.28 (1.09-4.77) | 3.73 (2.13-5.52) | 6.30 |
| **23A** | 71 | 28% | 0% | 30% | 28% | 14% | 6.32 (3.39-11.79) | 15.5% (8.9%-25.7%) | 1.53 (0.72-3.24) | 3.07 (1.71-4.66) | 4.90 |
| 6A | 80 | 34% | 1% | 38% | 25% | 3% | 7.54 (4.24-13.41) | 11.3% (6%-20%) | 1.2 (0.55-2.62) | 2.51 (1.37-3.81) | 4.20 |
| **10A** | 82 | 32% | 1% | 35% | 26% | 6% | 6.86 (3.84-12.26) | 18.3% (11.4%-28%) | 2.03 (1.03-4.01) | 3.92 (2.47-5.51) | 6.90 |
| **20** | 127 | 10% | 2% | 56% | 24% | 7% | 1.66 (0.86-3.21) | 8.7% (4.9%-14.8%) | 0.78 (0.39-1.59) | 1.88 (0.96-2.92) | 3.40 |
| **11A** | 140 | 11% | 2% | 44% | 31% | 11% | 1.91 (1.01-3.58) | 30% (23%-38%) | 3.77 (2.29-6.21) | 5.82 (4.4-7.38) | 10.30 |
| **33F** | 170 | 7% | 1% | 69% | 19% | 4% | 1.12 (0.57-2.19) | 10% (6.3%-15.4%) | 1.3 (0.71-2.37) | 1.95 (1.14-2.78) | 3.30 |
| 6B | 180 | 16% | 2% | 48% | 26% | 8% | 2.76 (1.66-4.6) | 11.1% (7.3%-16.5%) | 1.2 (0.68-2.11) | 2.29 (1.45-3.17) | 4.10 |
| **9N** | 184 | 15% | 2% | 55% | 22% | 6% | 2.56 (1.52-4.31) | 20.7% (15.4%-27.1%) | 2.32 (1.43-3.78) | 3.99 (2.94-5.07) | 6.90 |
| 18C | 198 | 24% | 2% | 44% | 25% | 6% | 4.17 (2.63-6.62) | 10.6% (7%-15.7%) | 1.45 (0.82-2.57) | 2.39 (1.63-3.24) | 4.40 |
| **19F** | 220 | 28% | 1% | 38% | 24% | 10% | 5.46 (3.53-8.44) | 21.4% (16.5%-27.2%) | 2.58 (1.64-4.07) | 4.34 (3.42-5.37) | 7.50 |
| 23F | 236 | 22% | 3% | 48% | 22% | 5% | 4.01 (2.57-6.25) | 11% (7.6%-15.6%) | 1.24 (0.74-2.07) | 2.24 (1.59-2.97) | 3.70 |
| **12F** | 367 | 17% | 2% | 57% | 19% | 5% | 2.78 (1.83-4.24) | 14.2% (11%-18.1%) | 1.8 (1.17-2.79) | 2.98 (2.32-3.67) | 5.20 |
| **22F** | 454 | 13% | 1% | 63% | 20% | 4% | 1.99 (1.3-3.04) | 10.8% (8.2%-14%) | 1.36 (0.88-2.11) | 2.11 (1.59-2.65) | 3.50 |
| 9V | 498 | 10% | 3% | 68% | 17% | 2% | 1.5 (0.98-2.3) | 9.2% (7%-12.1%) | 1.16 (0.75-1.78) | 1.91 (1.44-2.43) | 3.30 |
| 4 | 524 | 7% | 2% | 69% | 18% | 4% | 1.04 (0.66-1.63) | 10.5% (8.2%-13.4%) | 1.26 (0.83-1.91) | 2.17 (1.67-2.66) | 3.90 |
| 19A | 587 | 11% | 3% | 59% | 23% | 4% | 1.8 (1.19-2.73) | 14.5% (11.9%-17.6%) | 2.02 (1.36-3) | 2.77 (2.28-3.34) | 4.70 |
| 3 | 617 | 17% | 6% | 52% | 20% | 6% | 2.81 (1.92-4.11) | 19.3% (16.4%-22.6%) | 2.48 (1.72-3.59) | 3.77 (3.23-4.36) | 6.50 |
| 14 | 622 | 7% | 4% | 70% | 17% | 2% | 1 | 8% (6.1%-10.4%) | 1 | 1.58 (1.19-1.95) | 2.70 |
| **8** | 1138 | 6% | 5% | 72% | 15% | 2% | 0.86 (0.58-1.28) | 5.7% (4.5%-7.2%) | 0.73 (0.49-1.08) | 1.17 (0.93-1.43) | 2.00 |
| 7F | 1224 | 6% | 6% | 71% | 15% | 2% | 0.77 (0.52-1.15) | 4.4% (3.4%-5.7%) | 0.74 (0.49-1.12) | 0.95 (0.73-1.17) | 1.70 |
| 1 | 1966 | 1% | 16% | 69% | 13% | 1% | 0.13 (0.07-0.21) | 2.8% (2.2%-3.6%) | 0.55 (0.37-0.83) | 0.57 (0.42-0.71) | 1.00 |

Table 4 Outcome regression for other factors as serotype for the age group 5-64 years

|  | Meningitis | Mortality |
| --- | --- | --- |
| Sex |  |  |
| Female | 1 | 1 |
| Male | 0.9 (0.78-1.03) | 0.96 (0.84-1.11) |
| Year |  |  |
| 2002-2003 | 1 | 1 |
| 2003-2004 | 0.58 (0.4-0.84) | 0.86 (0.58-1.26) |
| 2004-2005 | 0.65 (0.46-0.93) | 0.85 (0.59-1.22) |
| 2005-2006 | 0.69 (0.49-0.97) | 0.83 (0.59-1.18) |
| 2006-2007 | 0.83 (0.6-1.15) | 0.64 (0.45-0.91) |
| 2007-2008 | 0.68 (0.49-0.94) | 0.66 (0.47-0.92) |
| 2008-2009 | 0.74 (0.54-1.02) | 0.58 (0.41-0.81) |
| 2009-2010 | 0.81 (0.59-1.11) | 0.55 (0.39-0.78) |
| 2010-2011 | 0.67 (0.48-0.94) | 0.6 (0.42-0.86) |
| Comborbidities |  |  |
| With co-morbidities (Charlson>0) | 1 | 1 |
| No co-morbidities (Charlson = 0) | 1.88 (1.62-2.18) | 0.43 (0.37-0.5) |
| Meningitis |  |  |
| No | NA | 1 |
| Yes | NA | 1.63 (1.33-2) |
| Socio-economics |  |  |
| Level 1 | 1 | 1 |
| Level 2 | 0.96 (0.79-1.16) | 0.88 (0.73-1.07) |
| Level 3 | 1.01 (0.83-1.23) | 0.72 (0.58-0.88) |
| Level 4 | 0.95 (0.77-1.18) | 0.74 (0.59-0.92) |
| Level 5 | 1.21 (0.98-1.49) | 0.7 (0.55-0.89) |
| Alcoholism |  |  |
| No | 1 | 1 |
| Yes | 0.84 (0.74-0.96) | 1.82 (1.66-2) |

Table 5 Outcomes for the individual serotypes with n>50 for the age group 65 years and over (serotypes not in any of the PCV vaccines are highlighted in bold, and serotypes which are not in PCV or PPV vaccines are highlighted in bold and underlined).

| serotype | n | Meningitis | Empyema | Pneumonia | Septicemia | Other | Odds meningitis | Mortality | Odds Mortality | QALY (disc) | QALY(undisc) |
| --- | --- | --- | --- | --- | --- | --- | --- | --- | --- | --- | --- |
| **10A** | 61 | 5% | 0% | 64% | 26% | 5% | 1.23 (0.36-4.25) | 37.7% (26.6%-50.3%) | 1.86 (1.06-3.25) | 2.87 (1.91-3.97) | 3.60 |
| **17F** | 63 | 6% | 2% | 68% | 22% | 2% | 2.77 (0.92-8.32) | 30.2% (20.2%-42.4%) | 0.99 (0.56-1.76) | 1.99 (1.25-2.7) | 2.40 |
| **15B** | 79 | 9% | 0% | 61% | 28% | 3% | 3.5 (1.45-8.43) | 24.1% (16%-34.5%) | 0.84 (0.48-1.45) | 1.98 (1.23-2.8) | 2.40 |
| **31** | 88 | 7% | 0% | 61% | 27% | 5% | 2.37 (0.93-6.02) | 39.8% (30.2%-50.2%) | 1.62 (1.01-2.58) | 2.95 (2.13-3.81) | 3.60 |
| **35F** | 88 | 13% | 2% | 49% | 31% | 6% | 5.51 (2.59-11.73) | 35.2% (26.1%-45.6%) | 1.35 (0.84-2.17) | 2.68 (1.86-3.47) | 3.30 |
| **38** | 97 | 3% | 0% | 61% | 35% | 1% | 1.2 (0.35-4.08) | 23.7% (16.4%-33.1%) | 0.67 (0.4-1.11) | 1.43 (0.87-2) | 1.70 |
| **15A** | 99 | 3% | 1% | 65% | 29% | 2% | 0.94 (0.28-3.2) | 30.3% (22.1%-40%) | 1.16 (0.73-1.86) | 2.06 (1.4-2.78) | 2.50 |
| **16F** | 110 | 5% | 1% | 67% | 25% | 2% | 2.63 (1.04-6.6) | 36.4% (28%-45.7%) | 1.39 (0.91-2.14) | 2.69 (1.93-3.49) | 3.30 |
| 6C | 143 | 10% | 2% | 53% | 29% | 6% | 4.05 (2.04-8.08) | 25.9% (19.4%-33.6%) | 0.91 (0.6-1.39) | 1.77 (1.29-2.23) | 2.10 |
| **20** | 145 | 7% | 1% | 68% | 23% | 1% | 2.76 (1.3-5.84) | 33.8% (26.6%-41.8%) | 1.25 (0.85-1.83) | 2.33 (1.72-2.94) | 2.80 |
| 18C | 157 | 10% | 1% | 62% | 25% | 3% | 3.51 (1.86-6.65) | 26.1% (19.9%-33.5%) | 0.93 (0.63-1.38) | 1.87 (1.38-2.34) | 2.30 |
| **23A** | 181 | 9% | 2% | 57% | 24% | 8% | 3.72 (1.98-7) | 32% (25.7%-39.2%) | 1.17 (0.82-1.66) | 2.38 (1.86-2.94) | 2.90 |
| 6A | 182 | 8% | 1% | 65% | 24% | 3% | 3.38 (1.72-6.65) | 34.6% (28.1%-41.8%) | 1.26 (0.89-1.79) | 2.28 (1.81-2.79) | 2.70 |
| **12F** | 199 | 6% | 3% | 65% | 22% | 4% | 1.84 (0.92-3.69) | 20.6% (15.6%-26.8%) | 0.68 (0.47-1) | 1.55 (1.13-2) | 1.90 |
| **11A** | 233 | 3% | 2% | 65% | 27% | 3% | 1.06 (0.46-2.46) | 39.1% (33%-45.4%) | 1.62 (1.19-2.2) | 2.71 (2.22-3.25) | 3.30 |
| 9N | 251 | 6% | 1% | 70% | 23% | 1% | 1.77 (0.92-3.4) | 33.9% (28.3%-39.9%) | 1.38 (1.01-1.87) | 2.55 (2.04-3.05) | 3.20 |
| **33F** | 274 | 5% | 1% | 64% | 29% | 1% | 1.67 (0.87-3.22) | 26.3% (21.4%-31.8%) | 0.93 (0.68-1.27) | 2.07 (1.65-2.54) | 2.60 |
| 19F | 314 | 5% | 1% | 54% | 34% | 6% | 1.66 (0.89-3.09) | 40.8% (35.5%-46.3%) | 1.77 (1.35-2.32) | 3.09 (2.63-3.55) | 3.80 |
| 4 | 417 | 3% | 1% | 72% | 23% | 1% | 0.94 (0.49-1.81) | 27.8% (23.7%-32.3%) | 1.02 (0.79-1.33) | 2.13 (1.78-2.48) | 2.60 |
| 6B | 427 | 5% | 2% | 64% | 26% | 3% | 1.77 (1.01-3.09) | 34.9% (30.5%-39.5%) | 1.37 (1.07-1.75) | 2.52 (2.21-2.86) | 3.10 |
| 1 | 489 | 2% | 6% | 74% | 17% | 1% | 0.45 (0.21-0.96) | 16.8% (13.7%-20.3%) | 0.62 (0.47-0.83) | 1.38 (1.12-1.68) | 1.70 |
| 7F | 584 | 4% | 3% | 70% | 21% | 1% | 1.11 (0.65-1.91) | 19.8% (16.8%-23.3%) | 0.76 (0.59-0.99) | 1.52 (1.26-1.81) | 1.90 |
| 23F | 622 | 5% | 0% | 66% | 26% | 3% | 2.06 (1.25-3.37) | 32.3% (28.7%-36%) | 1.12 (0.9-1.4) | 2.23 (1.96-2.5) | 2.70 |
| 9V | 650 | 2% | 1% | 71% | 24% | 2% | 0.55 (0.27-1.09) | 37.2% (33.6%-41%) | 1.48 (1.2-1.83) | 2.74 (2.44-3.05) | 3.40 |
| **8** | 694 | 4% | 2% | 70% | 22% | 2% | 1.27 (0.76-2.13) | 25% (22%-28.4%) | 0.92 (0.73-1.15) | 1.91 (1.66-2.19) | 2.30 |
| **22F** | 706 | 3% | 2% | 69% | 24% | 2% | 1.05 (0.6-1.83) | 28.3% (25.1%-31.8%) | 1.03 (0.83-1.29) | 2.07 (1.81-2.35) | 2.60 |
| 19A | 891 | 2% | 2% | 70% | 24% | 1% | 0.79 (0.45-1.38) | 30.1% (27.2%-33.2%) | 1.14 (0.93-1.4) | 2.08 (1.84-2.32) | 2.50 |
| 3 | 1009 | 3% | 2% | 66% | 28% | 1% | 1.03 (0.63-1.71) | 39.3% (36.4%-42.4%) | 1.75 (1.45-2.12) | 2.93 (2.68-3.19) | 3.60 |
| 14 | 1110 | 3% | 2% | 71% | 23% | 1% | 1 | 29.2% (26.6%-31.9%) | 1 | 1.99 (1.81-2.19) | 2.40 |

Table 6 Outcome regression for other factors as serotype for the age group 65 years and over

|  | Meningitis | Mortality |
| --- | --- | --- |
| Sex |  |  |
| Female | 1 | 1 |
| Male | 0.84 (0.69-1.02) | 1.21 (1.11-1.32) |
| Year |  |  |
| 2002-2003 | 1 | 1 |
| 2003-2004 | 1.28 (0.75-2.21) | 1.24 (0.98-1.55) |
| 2004-2005 | 1.19 (0.71-1.98) | 1.1 (0.89-1.36) |
| 2005-2006 | 0.96 (0.56-1.62) | 1.09 (0.88-1.35) |
| 2006-2007 | 1.41 (0.85-2.33) | 1.07 (0.86-1.32) |
| 2007-2008 | 1.5 (0.92-2.46) | 0.94 (0.76-1.17) |
| 2008-2009 | 1.21 (0.74-1.99) | 0.97 (0.79-1.2) |
| 2009-2010 | 1.28 (0.78-2.11) | 0.86 (0.7-1.07) |
| 2010-2011 | 1.35 (0.8-2.28) | 0.91 (0.73-1.15) |
| Comborbidities |  |  |
| With co-morbidities (Charlson>0) | 1 | 1 |
| No co-morbidities (Charlson = 0) | 2.51 (2.06-3.05) | 0.68 (0.61-0.74) |
| Meningitis |  |  |
| No | NA | 1 |
| Yes | NA | 1.27 (1.02-1.58) |
| Socio-economics |  |  |
| Level 1 | 1 | 1 |
| Level 2 | 0.92 (0.67-1.26) | 0.9 (0.79-1.02) |
| Level 3 | 1.28 (0.96-1.73) | 0.95 (0.84-1.09) |
| Level 4 | 1.21 (0.89-1.63) | 0.98 (0.86-1.12) |
| Level 5 | 1.41 (1.04-1.92) | 0.87 (0.75-1) |
| Alcoholism |  |  |
| No | 1 | 1 |
| Yes | 0.82 (0.52-1.28) | 1.7 (1.38-2.1) |
